# An Automatic AI‐Based Algorithm That Grades the Scalp Surface Exfoliating Process From Video Imaging. Application to Dandruff Severity and Its Validation on Subjects of Different Ages and Ethnicities

**DOI:** 10.1111/jocd.70013

**Published:** 2025-04-07

**Authors:** Frederic Flament, Ava Mondji, Chengda Ye, Zeneng Sun, Panagiotis‐Alexandros Bokaris, Benjamin Askenazi, Emmanuel Malherbe, Romain Roncin, Aldina Suwanto, Adrien Chretien, Maxime De Boni, Angeline Young, Bianca Maria Piraccini, Victoria Barbosa, Guive Balooch

**Affiliations:** ^1^ L'Oréal Research and Innovation Clichy France; ^2^ L'Oréal Research and Innovation Shanghai China; ^3^ Gleneagles Medical Centre Singapore; ^4^ Dermatology Unit IRCCS Azienda Ospedaliero‐Universitaria di Bologna Bologna Italy; ^5^ Department of Medical and Surgical Sciences Alma Mater Studiorum University of Bologna Bologna Italy; ^6^ Section of Dermatology University of Chicago Medicine Chicago Illinois USA

**Keywords:** age, clinical imaging, dandruff, ethnic groups, exfoliation, scalp

## Abstract

**Objectives:**

To evaluate the technical assets of a new imaging device that, wifi linked to a AI based algorithm, automatically grades in vivo the exfoliating process of the skin, taking dandruff as model.

**Material and Methods:**

The hand portable device comprises a camera that possibly uses three illuminating conditions (white LED diffused lamp, cross‐polarized white light and UVA rays). The learning phase of the algorithm was built on 3600 images of the vertex area of 234 subjects of different ages and three ethnicities with and without dandruff. This learning phase allowed 15 experts and dermatologists to score regarding a 6‐point atlas of dandruff severities, taken as reference. In a second validation phase, 460 images from 192 subjects of different ages and ethnic background/phototypes, were automatically analyzed by the AI based device, allowing to calculate the correlation between expert's assessments and the gradings provided by the device, and, as second indicator, to compute the Mean Average Error (MAE) between both variables.

**Results:**

The values were found significantly correlated (*r* ^2^ = 0.952; *p* < 0.001) with an overall MAE of 0.16 grading units, although presenting some differences according to ethnic background and phototypes (0.12–0.24).

**Conclusion:**

This new imaging device coupled with AI‐based analysis allows a valid, rapid, and easy determination of the scalp exfoliating process and may represent a complementary help in the diagnosis of dermatologists in some other scalp disorders. Its versatility, easy handling, and immediate AI‐based analysis suggest that it may be applied to other cosmetic areas (skincare, makeup, haircare, etc.).

## 1 Introduction

In its normal status, the adult human scalp surface (≈600 cm^2^) is a *terra incognita*, visually speaking, hidden by the presence of some 100 000–130 000 hair of various diameters (70–120 μm) emerging from their respective pilo‐sebaceous duct that additionally drains the sebum excretion. although, hardly visible to the eyes, the scalp is nevertheless a site where itch is frequently presented [1, 2]. Although this nociceptive feeling may be caused by external agents linked to hair grooming (hard brushing, exposure to very hot hair drier, use of harsh soaps, etc.), a constitutive status created by the presence of sebum and a specific resident microbiome, referred as the “Triad” (*Cutibacteria acnes* ex, *Staphylococci* spp and yeasts of the *Malassezia* genus) [3, 4]. The rather high level of excreted sebum (100–300 μg/cm^2^ or above) naturally fuels a scalp flora that possesses a lipophilic oriented metabolism (lipases, oxygenase's, peroxidases, etc.) that lead to a continuous modification of the constituents of the scalp biotope [5, 6, 7, 8, 9, 10, 11] and the release of irritating or pro‐inflammatory agents. Such events are therefore prone at inducing changes in the keratinization process of the scalp that, as compared to other skin sites, presents an increased turn‐over of up to 2 weeks [12, 13].

This scenario is typically the one found in dandruff (and its more severe form, the Seborrheic Dermatitis) where the keratinization process appears uncomplete and delivers a high proportion of squames, that is clumps (with a mean size of 233 μm) [14] formed by hundreds to thousands stacked immature corneocytes where nuclei remnants can be observed, that is a parakeratotic status linked to a sub‐inflammatory reaction [6, 12, 15], the likely source of itch as the latter has been closely linked with the severity of dandruff [6, 15].

The recent development of a hand portable camera equipped with three illuminating modes (white LED light, cross‐polarized white light, and UVA rays) seemed a good opportunity to explore its capacity in performing a precise status of the scalp, as a help to diagnosis for dermatologists or cosmetic purposes. With regard to dermatological observations, apart from onset of alopecia, it might well detect some “silent” scalp invaders (Demodex, Headlouse, etc.), visible indicators of bacterial infections (Impetigo, tinea capitis, folliculitis), etc…. The camera has a resolution of 3840 × 2160 pixels, with a field of view measuring 10.6 × 5.8 mm, resulting in a pixel size of approximately 0.00276 mm.

As a first step, its availability led us to explore its capacity to quantify the exfoliating process of the scalp in vivo, taking the dandruff condition as model. Physically speaking, dandruff squames are objects of a 0.5–4 mm size, that is visible, that adhere to the scalp and hair, and with time and head hair movements, some are transferred to garments. Nowadays, a large amount of works concurs to confer to the permanent presence of Malassezia yeasts (globosa, restricta, etc.) onto the scalp, one of the causative agent of dandruff in sensitive subjects predisposed to develop dandruff due to underlying scalp conditions [3, 5]. Such relationship is reinforced by the fact that the dandruff condition is easily and efficiently fought by topical application (shampoo form mostly) of ingredients that share a strong anti‐fungal property despite very different chemical structures (Selenium Sulphide, Piroctone Olamine, Ketoconazole, etc.). Although the use of these preparations once or twice a week (or more) systematically lead to a rapid decrease of itch and squames, its cessation slowly restores the *ante* condition within 2–3 weeks.

Grading the severity of dandruff, correlated to the density of squames, intensity of itch and parakeratotic status [6, 15] is not an easy procedure, mostly performed by clinical assessments of scalp adhering squames and those trapped by hair. Some objective methods have been developed [15]—vigorous scrubbing above an aluminum foil, collecting squames extracted by a bland shampooing further evaluated by weighing. These useful methods, albeit somewhat tedious, helped to estimate the average squames production on a two‐day wash period at about 20–30 mg per scalp in subjects with dandruff, compared to 3–5 mg in non‐dandruff subjects. To address these limitations, the electronic device used is wifi connected to a tablet that uses an AI based algorithm, provides high quality images of the scalp, allowing detailed analysis of any scalp area (squames density, redness, hair diameters, and density (N/cm^2^), etc.).

Trained through the collection of 3600 cross‐polarized images of the scalp associated with experts' and dermatologists' assessments (dataset collection and learning phase), we aim to check the relevance and accuracy of its supplied data when used on 460 cross‐polarized images of scalp from subjects of different ages, ancestries, phototypes, and different severities of dandruff (validation phase). Basically, the present study was conducted under a similar process of our previous works dealing with facial aging that automatically graded the severity of some skin signs, recorded from selfies images [16, 17, 18, 19, 20]. The results of this study are the objects of the present paper.

## 2 Material and Methods

### 2.1 The ScalpConsult Pro Device

**FIGURE 1:**
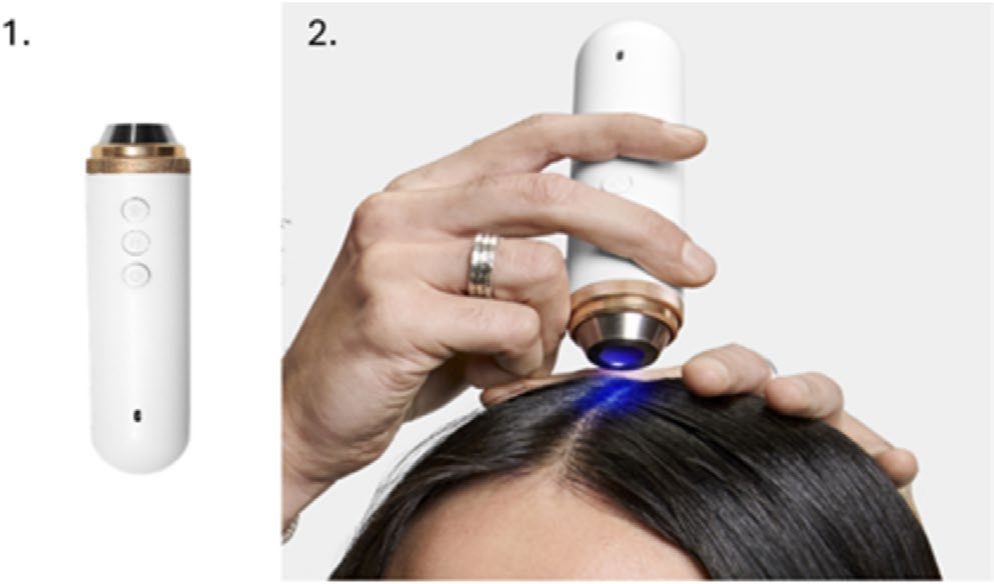
Photos 1 and 2 showing the view of 1. ScalpConsult Pro device (left) and 2. Its use on the scalp (right).

**FIGURE 2:**
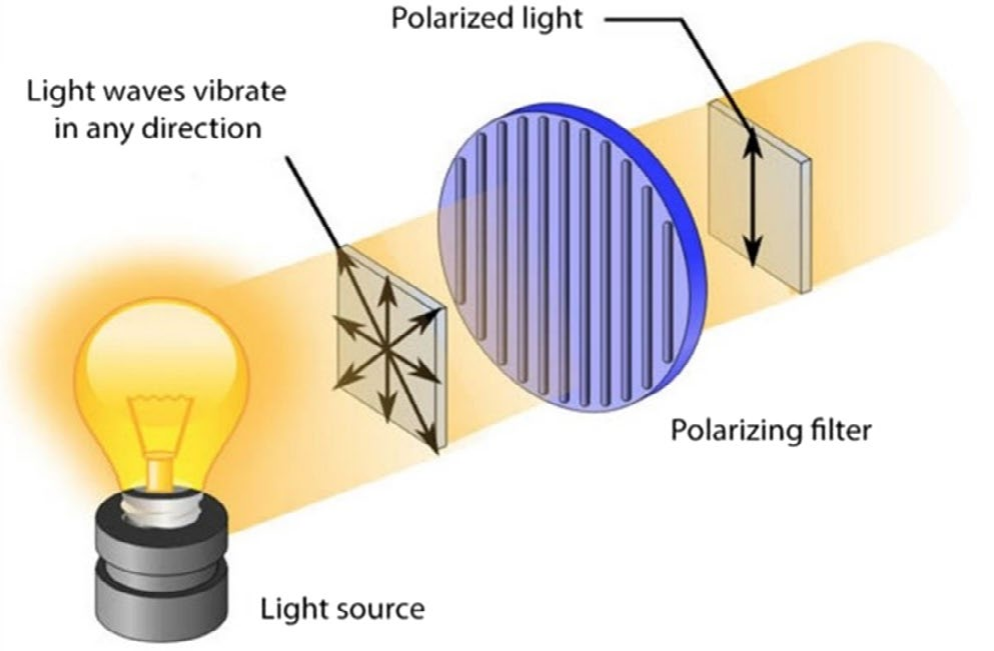
The illuminating system of the ScalpConsult Pro camera. Light source includes a UVA lamp.

**FIGURE 3:**
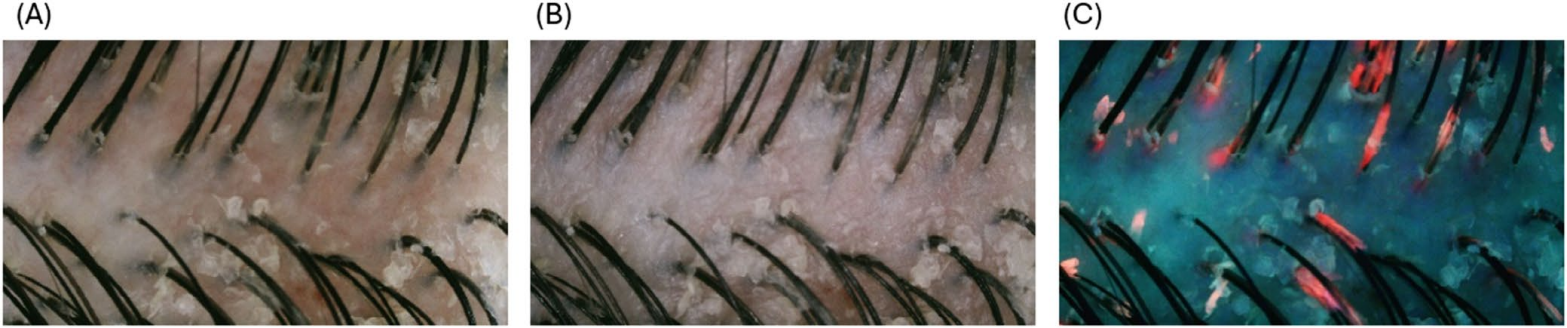
Photos 3 (A–C) (left to right): the images of the same dandruff scalp area (vertex), illuminated by the three different sources (A: white led diffused light, B: cross‐polarized light and C: UVA source, revealing the fluorescence of Porphyrins in orange/red).

This patented hand portable camera (weight: 120 g; length: 14 cm; see Figure 1), manufactured by Guangzhou Telefield Ltd. (Guangzhou, China), comprises three possible lighting modes (LED white diffused light, cross‐polarized light and UVA, see Figure 2) with a field of view of 10.6 × 5.8 mm of a 3840 × 2160 pixels resolution. White Led diffused light reveals surface features shine/sebum, hair fibers, and cross polarized light allows same features while removing most glaze, to better assess the skin color, degree of redness or tone evenness. UVA illumination reveals mostly Porphyrins synthetized and secreted by *Cutibacerium* spp [21, 22]. Figure 3 show, as examples, the same image of a dandruff scalp taken with the three illuminating sources (A–C).

### 2.2 Protocol

**FIGURE 4:**
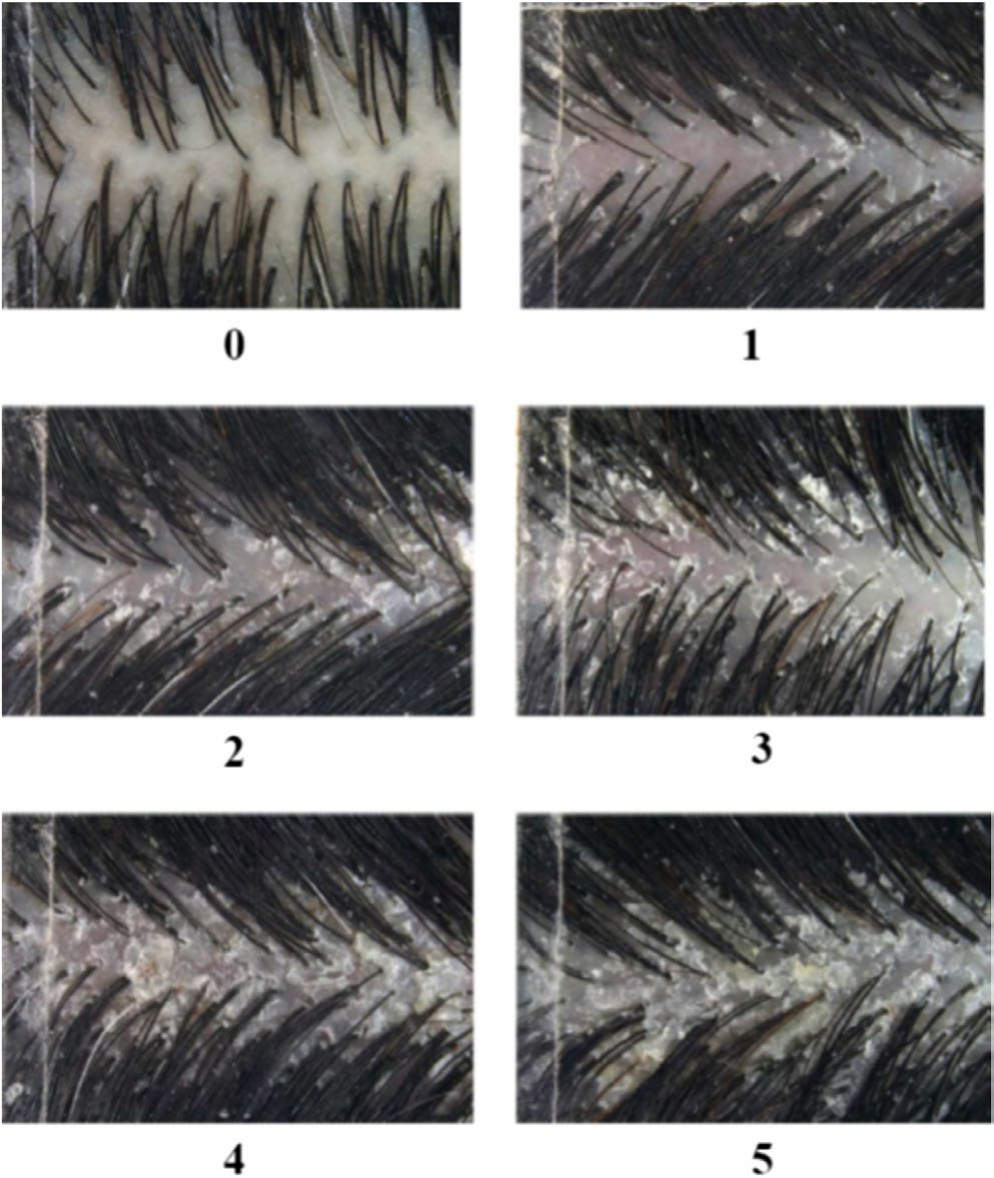
Six‐point standardized photographic scale describing severity of dandruff. Grade 0: absence; Garde 5: very severe.

The general protocol comprised a three phases process:

- *Dataset creation*: Informed written consent was obtained from all subjects after explaining the study procedures and their right to withdraw at any time. Given that no interventions on human subjects were carried out, no identifiable data was collected and only a scalp photo was taken; an ethics committee approval of the study protocol was not warranted.
  Two hundred and thirty‐four subjects of comparable dark hair color were recruited and seen in our two facilities that is, Paris/France (phototype I–III), Shanghai/China (phototype II–IV) and in a certified Clinical Research Organization located in Mauritius (phototypes IV–V). Subjects ranged from 18 to 60 years old, with 79% female and 21% male representation for the validation cohort. Clinical inclusion criteria targeted at least 10% of subjects with dandruff graded ≥ 3 based on a high‐dandruff clinical score and those exhibiting hair loss at ≥ 1–1 on the Ludwig scale (females) or ≥ III on the Hamilton scale (males). Other inclusion criteria included absence of any scalp pathology and being of healthy disposition, aged between 18 to 60 years. The study criteria also ensured a balanced number of men and women, ensuring diversity in gender representation across different phototypes. Subjects were asked to wash their hair twice a week 1 month before the visit, using a bland shampoo that was supplied and stopped washing their hair 3 days prior to shooting.
  Between 9 and 12 pictures were taken using ScalpConsult Pro device (cross‐polarized modality) before and after shampoo on vertex area for each volunteer, hence, 4060 images were collected globally. Fifteen trained experts and dermatologists have graded each image regarding a specific 6‐point standardized photographic scale presented in Figure 4 from absence to severe dandruff. The severity of dandruff was scored where grade 0: absence of dandruff to Garde 5: very severe. The process of grading has been previously described [23].
- *Learning phase*: ~90% of these images, 3600 cross‐polarized pictures, and their corresponding graded scores, evaluated by experts, were used to develop a dedicated AI‐based algorithm in a tablet permanently linked (wifi) with the ScalpConsult Pro camera, that automatically converts each image into a given grading score taken clinical scale (Figure 4) as reference. The distribution of the learning dataset by gender, age, and phototype is shown in Table 1.
- *Validation phase*: as ultimate step, 460 cross‐polarized images of the vertex area (~10%) were randomly chosen among the dataset of 4060 images representing a panel of 192 women and men of different ages (18–65 years), ancestries, phototypes and various severities of dandruff. The distribution of the validation dataset by gender, age, and phototype is shown in Table 2.

**TABLE 1:** Learning phase/distribution of the 234 subjects (*n*) and associated 3680 images according to countries/ethnicity, phototypes and ages.

Country (phototypes)	*n*	Women	Men	18–20 year	20–29 year	30–39 year	40–49 year	50–59 year	> 60 year	Images
China (II–IV)	105	68	37	0	9	35	34	24	3	2538
France (I–III)	99	89	10	3	17	23	31	22	3	1022
Mauritius (IV–V)	30	28	2	1	14	13	1	1	0	120

**TABLE 2:** Validation phase. Distribution of the 192 subjects (*n*) and associated 460 images according to countries/ethnicity, phototypes and ages.

Country (phototypes)	*n*	Women	Men	18–20 year	20–29 year	30–39 year	40–49 year	50–59 year	> 60 year	Images
China (II–IV)	93	61	32	0	6	33	30	22	2	259
France (I–III)	69	63	6	2	10	15	25	16	1	141
Mauritius (IV–V)	30	28	2	1	14	13	1	1	0	60

All blinded coded photos were sent to our secured website to being processed and further evaluated at random by the same 15 experts to be clinically graded and their related scores were averaged.

### 2.3 Statistics

- The paired correlations between experts' and dermatologists' grading versus AI‐based automatic scores used the Spearman's coefficient, taking a *p* < 0.05 value as threshold of statistical significance (SPSS software Package, Chicago, ILL, USA).
- As previously used [17, 18, 19, 20] privilege was given to calculate the Mean Absolute Error (MAE) as index of agreement between the data supplied by the AI‐based automatic grading system and those issued from the 15 experts and dermatologists, taken as gold standards. The difference between both data can be better expressed as the fractions (in %) where MAE is inferior to 0.1, 0.2, etc… up to 3.0 grading units.

## 3 Results

### 3.1 Correlation Coefficient

**FIGURE 5:**
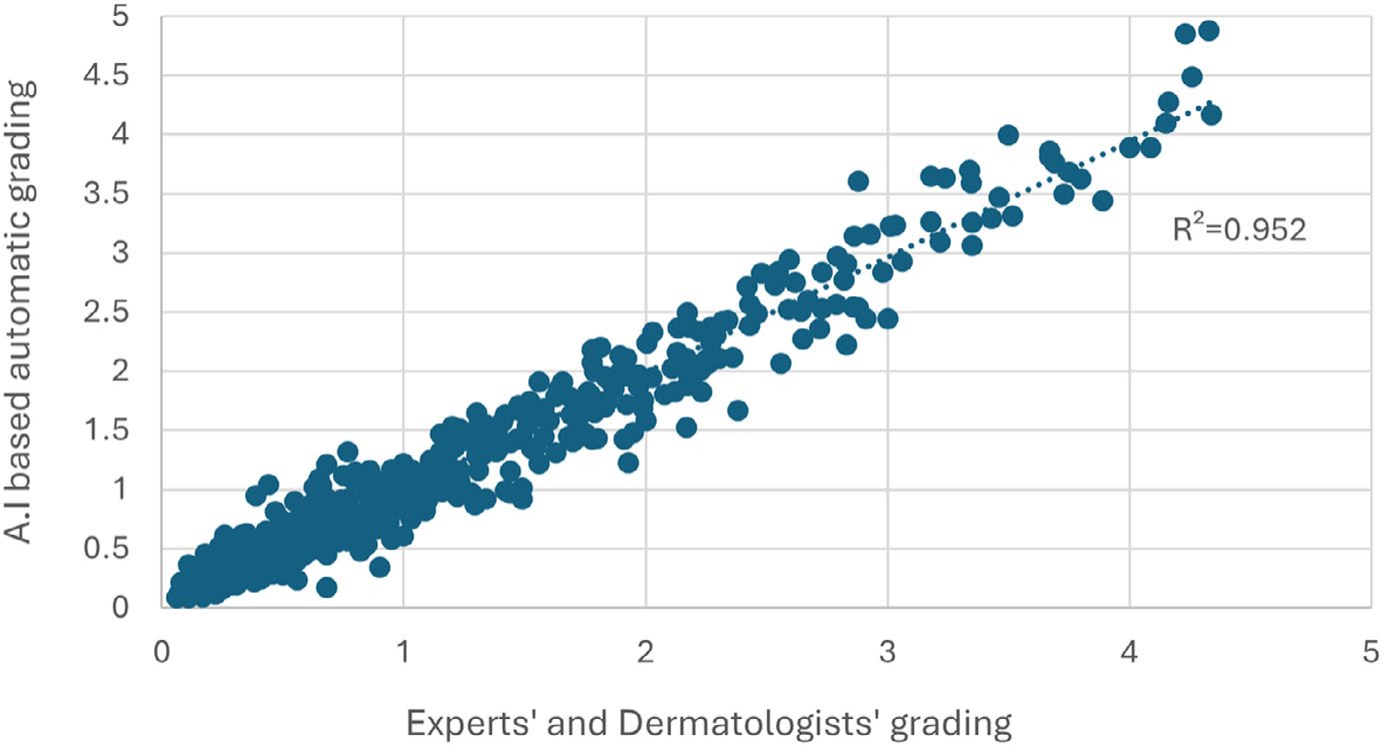
For all 460 images of validation phase correlation among experts' gradings and automatic severity scores.

Figure 5 shows the significant correlation (*r* ^2^ = 0.952; *p* < 0.001) between the grades provided by the ScalpConsult Pro device and those evaluated by the 15 experts and dermatologists for the 460 images dedicated to validation phase. By analyzing each ethnicity significant coefficients could be observed (*p* < 0.001) for France (*r* ^2^ = 0.945), China (*r* ^2^ = 0.943) and Mauritius (*r* ^2^ = 0.933).

### 3.2 Performance of AI‐Based Automatic Dandruff Severity Grading

As previously mentioned, calculating the Mean Average Errors (MAE = Δ average experts' gradings—AI‐based automatic gradings) on the 460 photos, allows to observe that in a large majority (≈85%), MAE do not exceed 0.3 grading units, among which 70% are inferior to 0.2 grading unit. Inversely, some 14% present average errors ranging 0.3–1 grading units (Table 3). In overall (Table 4), when gathering MAE within each ethnicity, some differences are observed, where errors from the subjects of Mauritius are about twice higher than those from the Chinese cohort (Table 5). In short, an overall value of 0.163 seems an acceptable and relevant value of average errors.

**TABLE 3:** Numbers of photos per MAE ranges (% as rounded values).

Number of images	MAE	% of the 460 images
189	< 0.1	41.0
136	0.1–0.2	29.5
68	0.2–0.3	14.7
37	0.3–0.4	8.0
15	0.4–0.5	3.2
15	0.5–1.0	3.2
0	1.0–1.5	0
0	1.5–2.0	0
0	2.0–3.0	0
0	> 3.0	0

**TABLE 4:** Overall MAE (grading units, rounded values) in the three studied ethnicities.

	Dandruff score	MAE
Total	≥ 3.0	0.24
	< 3.0	0.16
China (II–IV)	≥ 3.0	0.19
	< 3.0	0.12
France (I–III)	≥ 3.0	0.23
	< 3.0	0.20
Mauritius (IV–V)	≥ 3.0	0.32
	< 3.0	0.22

**TABLE 5:** Overall, MAE (grading units, rounded values).

Populations	MAE
Overall	0.16
China (II–IV)	0.13
France (I–III)	0.20
Mauritius (IV–V)	0.24

## 4 Discussion

The preliminary study presented here, focusing first on the exfoliating process of dandruff, indicates that the new ScalpConsult Pro device seems of a high interest, as a help to dermatologists and cosmeticians. Easy to handle, light and versatile, it provides clear images with different modalities (cross‐polarized, diffused, and UV lights), rapidly analyzed by an AI‐based automatic grading algorithm, provided a fully learning phase adapted and based upon clinical assessments, taken as gold standards.

In the case of dandruff, the good correlation of its automatic gradings between the experts' assessments and an acceptable mean average error, allows us to proceed to further fine tunings of the algorithm such as the possible impacts of the skin color (phototypes) or gender as the studied subjects were feminine in a large majority (152 out of 192). Some further studies are however needed to record all possible factors that influence the automatic analytical process as well as increasing the dataset in darker phototypes and so reduce MAE of 0.24 closer to average of 0.16 (Table 4). As example, the sizes of squames could present some variations (not studied here) according to ethnicity, age, or clinical severity. Accordingly, a larger study is currently undertaken, involving more than 20 000 subjects from 11 countries to better estimate all factors where improvements can and have to be brought. The present study did not arbitrarily consider the density of hair‐adhering squames (although detected by the camera). We estimate that these represent secondary elements of desquamation, that is, too much impacted by unavoidable external events (hair brushing, head hair movements, touches, wind, hair surface status, etc.), that is, not reflecting a genuine exfoliative process.

Nevertheless, with regard to dandruff, these preliminary promising results obviously authorizes further in vivo studies such as the follow up of an efficient anti‐dandruff shampoo with time [24]. Association of magnified images of scalp and ad‐hoc automatic analysis was previously discussed in literature [25]. Coupled with the recordings of sensorial assessments (like itching) of the tested subjects could allow to determine the automatic grading that corresponds to the threshold of their nociceptive feelings or to record the lingering effect of the treatment phase.

Versatility, easiness of handling, rapid acquisition, and accurate automatic analysis of images are paramount factors in a daily research routine. As promising help to dermatologists, such a methodology could well concur to diagnose or treat several scalp affections (psoriasis, male or female alopecia, alopecia areata, ringworms, tinea capitis, etc.) in a short time, easily collecting and saving individual images of a given subject. Although acne never involves the scalp, the use of the UVA lamp, by detecting the decreased presence of porphyrins, could well be used as a clinical marker of a topical bactericidal action or an oral antibiotic treatment targeting *Cutibacteria* spp. colonization, the major causative bacterial agent of acneic lesions.

The ScalpConsult Pro device represents an advancement over traditional scoring methods [3]. While the traditional method provides a global score for dandruff using a combination of visual and clinical expertise, the ScalpConsult Pro method introduces a more precise, localized, and reproducible approach to scalp evaluation. By using high‐resolution imaging under three different lighting conditions (polarized, cross‐polarized, and natural light), this device allows for a detailed analysis of individual scalp regions, capturing subtle variations in dandruff severity and distribution that could have been overlooked in broader, visually estimated scores. Furthermore, a traditional atlas‐based scoring relies on subjective judgment and averages across large scalp areas while the ScalpConsult Pro focuses on consistently imaged regions which enhances repeatability and objectivity.

With regards to cosmetic applications, where large cohorts are often needed in vivo, this methodology offers both nomadic and holistic approaches. After dandruff clinical severity the addition of other automatic algorithms to assess key scalp descriptors like hair density, hair thickness or standard deviation of hair thickness could be a pathway for more holistic diagnostic of scalp health. Based on internal data a link between a combination of these parameters will be established with well‐being and emotional state of women and men worldwide. The use of the device on other body sites or modified annexes (hair, nails, eyebrows, etc.) is another domain to be explored, needing first the development of specific, robust, and relevant algorithms. In short, this device and its associated automatic analytic process appear much extending the arsenal of many technologies that validate or claim the effects of cosmetic products and procedures.

A limitation reported from this study included limited ability of the algorithm to generalize across different populations. To address this, the training dataset could be expanded to include a more diverse set of images, with specific focus on ethnicities and phototypes which could improve the algorithm's ability to grade across different populations. Adjusting the algorithm by integrating parameters tailored to distinct scalp and skin characteristics observed in different ethnic groups could also be explored. Finally, additional validation will be conducted on datasets specific to ethnicity and phototype.

For the MAE by phototype (Table 5), the values are higher for the Mauritian population, likely due to the limited representation of darker phototypes in the dataset. Increasing the number of images of darker phototypes in future studies may help reduce the MAE and improve overall accuracy. As future directive, we also plan to refine the dataset by incorporating hair curliness as an additional parameter.

Another limitation of the validation dataset was an imbalance in gender representation, with a majority of female subjects. This raises concerns about potential biases in the algorithm's performance, particularly if gender‐specific factors, such as differences in scalp physiology and hair density affect the accuracy of scoring. While this preliminary analysis does not report gender‐related discrepancies in algorithm performance, further investigation is necessary to confirm this finding.

Finally, as a future directive, applying similar automatic AI‐algorithm tools to other dermatological conditions, such as acne, psoriasis or melasma, could highlight its versatility in skin health diagnostics and treatment planning. These would demonstrate the potential of automatic AI‐algorithms to improve diagnostic accuracy and also offer personalized treatment. Therefore, situating this study within a broader context offer a directive for future investigations to explore AI's applicability in a wider range of dermatological challenges.

## Author Contributions

F.F. and A.M. were involved in the design, conduct, collection, management, analysis, interpretation preparation and decision to submit the manuscript for publication; they had full access to all the data in the study and takes responsibility for the integrity, of the data and the accuracy of the data analysis. C.Y., Z.S., E.M., A.B. and B.A. created the AI‐based software for grading and simulation. R.R., A.S. and A.C. were involved in the design of the work, in interpretation and analysis of results. G.B., A.Y., B.M.P., V.B. made a substantial contribution in drafting and revising the manuscript. All authors reviewed and approved the manuscript before submission and have agreed to be accountable for their contributions.

## Conflicts of Interest

F.F., A.M., C.Y., Z.S., A.B., B.A., E.M., R.R., A.S., A.C. and G.B. are employees of the L'Oréal group. A.Y., B.M.P., and V.B. have received grants and honorarium from L'Oréal.

## Data Availability

The data that support the findings of this study are available from the corresponding author upon reasonable request.
